# Self-Sustained Euler Buckling of an Optically Responsive Rod with Different Boundary Constraints

**DOI:** 10.3390/polym15020316

**Published:** 2023-01-07

**Authors:** Dali Ge, Yuntong Dai, Kai Li

**Affiliations:** 1 School of Civil Engineering, Anhui Jianzhu University, Hefei 230601, China; 2 Institute of Advanced Technology, University of Science and Technology of China, Hefei 230001, China

**Keywords:** self-sustained oscillation, Euler buckling, liquid crystal elastomers, optically responsive, rod

## Abstract

Self-sustained oscillations can directly absorb energy from the constant environment to maintain its periodic motion by self-regulating. As a classical mechanical instability phenomenon, the Euler compression rod can rapidly release elastic strain energy and undergo large displacement during buckling. In addition, its boundary configuration is usually easy to be modulated. In this paper, we develop a self-sustained Euler buckling system based on optically responsive liquid crystal elastomer (LCE) rod with different boundary constraints. The buckling of LCE rod results from the light-induced expansion and compressive force, and the self-buckling is maintained by the energy competition between the damping dissipation and the net work done by the effective elastic force. Based on the dynamic LCE model, the governing equations for dynamic Euler buckling of the LCE rod is formulated, and the approximate admissible trigonometric functions and Runge-Kutta method are used to solve the dynamic Euler buckling. Under different illumination parameters, there exists two motion modes of the Euler rod: the static mode and the self-buckling mode, including alternating and unilateral self-buckling modes. The triggering conditions, frequency, and amplitude of the self-sustained Euler buckling can be modulated by several system parameters and boundary constraints. Results indicate that strengthening the boundary constraint can increase the frequency and reduce the amplitude. It is anticipated that this system may open new avenues for energy harvesters, signal sensors, mechano-logistic devices, and autonomous robots.

## 1. Introduction

Self-sustained oscillations are out-of-equilibrium phenomena arising from built-in negative feedback loops [1], which can directly absorb the energy from the constant environment to maintain its periodic motion by self-regulating, such as cell division, heartbeats, neural impulses, and circadian clocks. The frequency and amplitude of self-oscillation only depend on the inherent properties of the system, and the self-oscillation has no requirement for additional complex controllers or heavy batteries [2,3]. In addition, self-sustained oscillation generally has good robustness [4], and the stability and normal operation of various systems can be ensured based on self-sustained oscillation. If human-made self-oscillations could be generated through life-like mechanisms and powered by constant environmental sources, it would be extremely advantageous for robotics, especially autonomous robots [5,6,7]. Moreover, due to its remarkable advantages, self-oscillating systems have potential application in energy harvesters [8], self-propelling devices [9], mass transport devices [10], signal sensors [11], mechano-logistic devices [12], and biomimetic designs [13].

In recent years, various self-oscillating systems based on different stimuli-responsive materials have been constructed, such as thermally responsive polymer materials [14], hydrogels [15,16], ion gels [17], dielectric elastomer [18], liquid crystal elastomers (LCEs) [19,20], etc. Meanwhile, a variety of self-sustained motion modes have been proposed, such as rolling [4,7,21], bending [22,23], vibration [24,25], torsion [25,26], stretching and shrinking [27,28], spinning [29], swinging [30], buckling [31,32,33,34], jumping [35,36,37], rotation [38], snap-through [39], eversion or inversion [40], expansion and contraction [26,41,42], swimming [43,44], and even group behavior coupled by two asymmetric oscillators [45]. In order to compensate for the energy loss of self-sustained oscillation caused by damping dissipation, different self-feedback mechanisms have been proposed, such as self-shadowing mechanism [41], coupling mechanism among chemical diffusion, large deformation and chemical reaction [46], and multi process coupling mechanism among droplet evaporation, motion, and plate buckling [47].

Light is an excitation with the unique advantages of contactless driving, programmability, ecological efficiency, and high temporal and spatial resolution, and has broad application prospects in various fields [48,49]. LCE is an important optically responsive material, which is synthesized by anisotropic rod-like liquid crystal molecules and stretchable long-chain polymers [50]. The deformation of LCE is determined by the arrangement of liquid crystal molecules in the elastic network. Under external stimuli including ultraviolet (UV) light, irradiation, electricity, magnetic fields, and heat [51], the cross-linked liquid crystalline polymer can be transformed between liquid crystalline and isotropic phases. Especially stimulated by light, LCE has the advantages of fast response and large and recoverable deformation, which is more convenient to induce customized feedback in various ways to achieve the light-induced self-sustained oscillation. Thus, light-fueled self-oscillating systems based on LCE have attracted wide attention, and many light-fueled self-sustained oscillation systems based on LCE have been constructed, such as torsion [25], jumping [35,36], and vibration [52].

To meet the multi-functional needs of autonomous robots, more modes of self-sustained oscillation systems still need to be developed. The rod is an important component widely used in aerospace aircrafts, robots, precision instruments, and other structures [53,54]. As a classical mechanical instability phenomenon, the Euler buckling of a compression rod can be used as a new way to produce reliable self-sustained oscillation [55], and several experimental works on light-driven buckling of LCE have been reported in Refs. [31,32,33], recently. In this paper, we propose a new self-sustained Euler buckling LCE rod under steady illumination and investigate the effects of five typical boundary configurations on the oscillation. The Euler compression rod can release elastic strain energy during buckling, and has the advantages of rapid energy release and large displacement. In addition, its boundary configuration is usually easy to be modulated; thus, the self-sustained Euler buckling LCE rod has potential application prospects in the fields of jumping robot, rescue, military industry, mechanical logic, and so on. 

The layout of this paper is as follows. In Section 2, the governing equations for dynamic Euler buckling of a LCE rod are first formulated based on dynamic LCE model, then the approximate admissible trigonometric functions and Runge–Kutta method are used to solve the dynamic Euler buckling of the LCE rod under steady illumination. In Section 3, two motion modes of the LCE rod under steady illumination are numerically solved and the mechanism of its self-sustained Euler buckling is revealed in detail. In Section 4, the effects of two typical system parameters on the triggering conditions, frequency and amplitude of the self-sustained Euler buckling, are studied. In Section 5, the effects of boundary configurations on self-sustained Euler buckling are discussed extensively. Finally, the conclusion is given in Section 6.

## 2. Theoretical Model and Formulation

### 2.1. Governing Equations for Dynamic Euler Buckling of a LCE Rod

**Figure 1:**
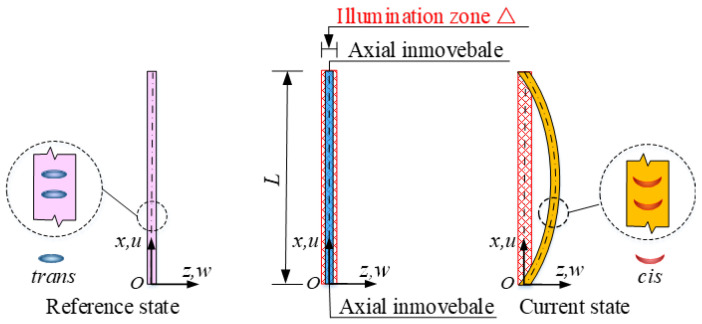
Schematics of an optically responsive LCE rod with length L, cross-section area A, and area moment of inertia J, which is capable of self-sustained Euler buckling under steady illumination with narrow zone Δ near its static equilibrium position. Its two ends are considered to be immovable in the axial direction. The azobenzene liquid crystal molecules in the rod are perpendicular to its axis and transform from straight *trans* to bent *cis* under illumination, giving rise to the rod’s shrinkage in the transverse direction and expansion in the axial direction.

Figure 1 sketches an optically responsive LCE rod with length L, cross-section area A, and area moment of inertia J. A coordinate system is introduced, where the coordinate origin O is at the lower endpoint of the rod, x and z are the axial coordinate and lateral coordinate, respectively. A narrow zone Δ near static equilibrium position of LCE rod is illuminated by a steady light, and the other zone is non-illuminated. The optically responsive azobenzene liquid crystal molecules [55,56,57,58] in the LCE rod are perpendicular to its axis, when exposed to the illumination, they transform from straight *trans* state to bent *cis* state, leading the rod to shrink in the transverse direction and expand in the axial direction. In this paper, the two rod ends are considered to be immovable in the axial direction. Once the light-induced expansion exceeds a critical value, the thin rod may buckle into the non-illuminated zone. Then, the light-induced expansion of the rod decreases with time, and the rod tends to move back to the illumination zone. When the rod enters the illumination zone again, its light-induced expansion increases with time, which may enable the thin rod to buckle to another side of static equilibrium position due to its own inertia. Therefore, the LCE rod under constant illumination eventually buckles alternately and periodically in both sides of the static equilibrium position. To focus on self-sustained Euler buckling, we assume that the rod width is much larger than its thickness, and the rod buckles always in the same xOz plane in the same way for perfect buckling. The non-linear strain displacement relations of the rod are given by [59]

(1) $$\varepsilon_{x}(x,t)=u_{,x}+\frac{1}{2}{\left(w_{,x}\right)}^{2},$$

(2) $$\psi_{x}(x,t)=w_{,xx},\;$$

where u(x,t) and w(x,t) are the axial displacement and lateral displacement at point x on the axis at time t, respectively. 

For an elastic material obeying Hooke’s law, we have,

(3) $$\sigma (x,t)=E\left(\varepsilon_{x}-\varepsilon_{L}\right),$$

Where σ(x,t) is the normal stress, E is the Young’s modulus, and $\varepsilon_{L}\left(t\right)$ is the light-induced expansion.

Considering that the azobenzene liquid crystal molecules are perpendicular to the length in the slender LCE rod, the rod under illumination expands in the length and contracts in the transverse direction. For a narrow illumination zone, we assume that the rod always enters or leaves the narrow illumination zone as a whole at the same time, and thus the light-induced expansion $\varepsilon_{L}\left(t\right)$ is uniform in the rod. Following Refs. [58,60], the light-induced expansion $\varepsilon_{L}\left(t\right)$ changes with time depending on the number fraction ϕ(t) of *cis*-isomers of LCE material. For simplicity, the light-induced expansion $\varepsilon_{L}\left(t\right)$ is assumed to be linearly related to ϕ(t) [50], i.e.,

(4) $$\varepsilon_{L}(t)=C_{0}\phi (t),$$

where $C_{0}$ is the expansion coefficient. The number fraction ϕ(t) will be given in Section 2.2.

Through the above assumptions and omitting the effect of shear deformation, the strain energy U and kinetic energy K of the LCE rod can be given as follows [61]

(5) $$U=\frac{EA}{2}\int_{0}^{L}\varepsilon_{x}^{2}dx+\frac{EJ}{2}\int_{0}^{L}\psi_{x}^{2}dx,$$

(6) $$K=\frac{\rho A}{2}\int_{0}^{L}w_{,t}^{2}dx.$$

It is worth noting that the effect of gravity on rod buckling can be neglected due to its weak influence. Assuming that the damping force is proportional to the velocity $w_{,t}$ and the light-induced load is denoted as $P_{L}=EA\varepsilon_{L}$, the virtual work of load can be given by [61]

(7) $$\delta W_{F}=P_{L}\int_{0}^{L}w_{,x}\delta w_{,x}dx-\int_{0}^{L}\beta w_{,t}\delta wdx,$$

where ρ is the mass density, and β is the damping coefficient.

The governing equation for the dynamic buckling of the rod can be expressed by the Hamilton’s theorem [62]

(8) $$\int_{0}^{t}\left(\delta U-\delta K-\delta W_{F}\right)dt=0.\;$$

Substituting Equations (1), (2) and (5)–(7) into Equation (8) for variational and integral operations, the governing equation of LCE rod can be derived as

(9) $$EA\varepsilon_{x,x}=0,\;$$

(10) $$EJw_{,xxxx}-EA{\left(\varepsilon_{x}w_{,x}\right)}_{,x}+EA\varepsilon_{L}w_{,xx}+\rho Aw_{,tt}+\beta w_{,t}=0,\;$$

where $w_{,tt}$ is the second-order differentiation with respect to t.

### 2.2. Evolution of the Number Fraction in the LCE Rod

To investigate the dynamic Euler buckling of the LCE rod, its number fraction of *cis*-isomers in Equation (4) should be calculated. The experimental results show that the *trans*-to-*cis* isomerization of LCE could be induced by UV or laser with wavelength less than 400 nm [63] and by blue light with wavelength 465 nm [64]. It is noted that the temperature increase caused by irradiation can also induce deformation because the nematic order monotonically decreases with temperature. For simplicity, we neglect the effect of the temperature increase, and only consider the pure molecular effect of photoisomerization [54,58]. The number fraction ϕ(t) of the *cis*-isomer is determined by the thermal excitation from *trans* to *cis*, the thermally driven relaxation from *cis* to *trans*, and the light driven relaxation from *trans* to *cis*. The number fraction ϕ(t) can be described by the well-established dynamic LCE model as [50].

(11) $$\phi_{,t}=\eta_{0}I_{0}\left(1-\phi \right)-T_{0}^{-1}\phi ,$$

where $T_{0}$ is the thermal relaxation time from *cis* state to *trans* state, $I_{0}$ is the light intensity, and $\eta_{0}$ is the light absorption constant. 

In the illumination zone, ϕ(t) can be obtained by solving Equation (11) as

(12) $$\phi \left(t\right)=\frac{\eta_{0}T_{0}I_{0}}{\eta_{0}T_{0}I_{0}+1}+\left(\phi_{0}-\frac{\eta_{0}T_{0}I_{0}}{\eta_{0}T_{0}I_{0}+1}\right)\exp\left[-\left(\eta_{0}T_{0}I_{0}+1\right)\frac{t}{T_{0}}\right],$$

where $\phi_{0}$ is the number fraction of *cis*-isomers at the initial moment under illumination.

In this paper, the LCE rod switches between illumination zone and dark zone. For Case I in which the LCE rod in the illumination zone with initial $\phi_{0}=0$, Equation (12) can be reduced to

(13) $$\phi \left(t\right)=\frac{\eta_{0}T_{0}I_{0}}{\eta_{0}T_{0}I_{0}+1}\left\{1-\exp\left[-(\eta_{0}T_{0}I_{0}+1)\frac{t_{1}}{T_{0}}\right]\right\}.$$

For Case II in which the LCE rod in the illumination zone switches from dark zone with transient $\phi_{0}=\phi_{\mathrm{dark}}$, Equation (12) can be reduced to

(14) $$\phi \left(t\right)=\frac{\eta_{0}T_{0}I_{0}}{\eta_{0}T_{0}I_{0}+1}+\left(\phi_{\mathrm{dark}}-\frac{\eta_{0}T_{0}I_{0}}{\eta_{0}T_{0}I_{0}+1}\right)\exp\left[-(\eta_{0}T_{0}I_{0}+1)\frac{t_{2}}{T_{0}}\right].$$

For Case III in which the LCE rod in the dark zone ($I_{0}=0$) switches from illumination zone with transient $\phi_{0}=\phi_{\mathrm{illum}}$, Equation (12) can be reduced to

(15) $$\phi \left(t\right)=\phi_{\mathrm{illum}}\exp\left(-\frac{t_{3}}{T_{0}}\right),\;$$

where $t_{1}$, $t_{2}$, and $t_{3}$ are the durations of current process, respectively, $\phi_{\mathrm{dark}}$ and $\phi_{\mathrm{illum}}$ are the number fractions of *cis*-isomers at the moment of switching from dark zone into illumination zone and from illumination zone into dark zone, respectively.

### 2.3. Nondimensionalization 

To conveniently investigate the dynamic buckling of the LCE rod, the dimensionless quantities are introduced as follows: $\bar{x}=x/L$, $\bar{w}=w\sqrt{A/J}$, $\bar{u}=uAL/J$, $\tau_{0}=\sqrt{\rho L^{4}A/EJ}$, $\bar{t}=t/\tau_{0}$, $\bar{\beta }=\beta \sqrt{L^{4}/\rho AEJ}$, ${\bar{I}}_{0}=\eta_{0}T_{0}I_{0}$, ${\bar{T}}_{0}=T_{0}/\tau_{0}$, ${\bar{\varepsilon }}_{L}=\varepsilon_{L}L^{2}A/J$, and $\varepsilon_{0}=C_{0}{\bar{I}}_{0}L^{2}A/\left[\left({\bar{I}}_{0}+1\right)J\right]$. Here, $\tau_{0}$ is the natural vibration characteristic time of the LCE rod. Generally, the larger the dimensionless damping coefficient $\bar{\beta }$ is, the faster the damping energy dissipation is. The dimensionless thermal relaxation time ${\bar{T}}_{0}$ represents the ratio of the *cis*-to-*trans* thermal relaxation time relative to the characteristic time $\tau_{0}$. The larger ${\bar{T}}_{0}$ is, the slower the *cis*-to-*trans* conversion is. The value of the dimensionless light-induced expansion loading $\varepsilon_{0}$ is determined by both the square of slenderness ratio $L^{2}A/J$ of the rod and maximum light-induced expansion $C_{0}{\bar{I}}_{0}/\left({\bar{I}}_{0}+1\right)$ in the illumination zone.

The governing Equations (9) and (10) can also be rewritten in the dimensionless form as

(16) $${\bar{u}}_{,\bar{x}\bar{x}}+{\bar{w}}_{,\bar{x}}{\bar{w}}_{,\bar{x}\bar{x}}=0,\;$$

(17) $${\bar{w}}_{,\bar{x}\bar{x}\bar{x}\bar{x}}=\left[{\bar{u}}_{,\bar{x}}+\frac{1}{2}{\left({\bar{w}}_{,\bar{x}}\right)}^{2}\right]{\bar{w}}_{,\bar{x}\bar{x}}-{\bar{\varepsilon }}_{L}{\bar{w}}_{,\bar{x}\bar{x}}-{\bar{w}}_{,\bar{t}\bar{t}}-\bar{\beta }{\bar{w}}_{,\bar{t}}.$$

**Figure 2:**
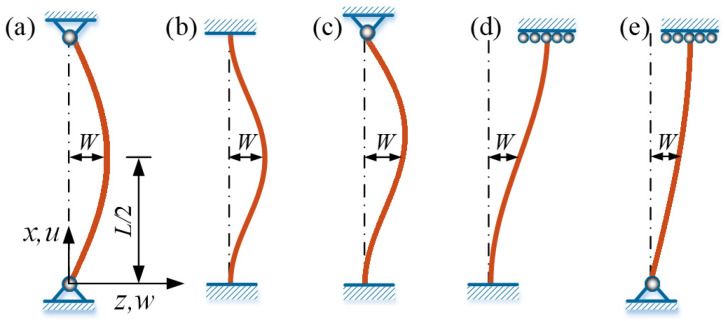
The five typical Euler buckling cases. (**a**) hinged-hinged (H-H); (**b**) clamped-clamped (C-C); (**c**) clamped-hinged (C-H); (**d**) clamped-guided (C-G); (**e**) hinged-guided (H-G). The guided boundary condition G represents that the lateral displacement is not constrained but the rotation is constrained.

In the present study, five typical buckling configurations of the Euler rod are considered: hinged-hinged (H-H), clamped-clamped (C-C), clamped-hinged (C-H), clamped-guided (C-G), and hinged-guided (H-G), and the schematic diagram is shown in Figure 2. The guided boundary condition G represents that the lateral displacement is not constrained, but the rotation is constrained. The boundary conditions for the governing Equations (16) and (17) are

(18) $$\mathrm{Case}\;H-H:\;\bar{w}={\bar{w}}_{,\bar{x}\bar{x}}=0\;\mathrm{both}\;\mathrm{at}\;\bar{x}=0\;\mathrm{and}\;\bar{x}=1,\;$$

(19) $$\mathrm{Case}\;C-C:\;\bar{w}={\bar{w}}_{,\bar{x}}=0\;\mathrm{both}\;\mathrm{at}\;\bar{x}=0\;\mathrm{and}\;\bar{x}=1,\;$$

(20) $$\mathrm{Case}\;C-H:\;\bar{w}={\bar{w}}_{,\bar{x}}=0\;\mathrm{at}\;\bar{x}=0\;\mathrm{and}\;\bar{w}={\bar{w}}_{,\bar{x}\bar{x}}=0\;\mathrm{at}\;\bar{x}=1,\;$$

(21) $$\mathrm{Case}\;C-G:\;\bar{w}={\bar{w}}_{,\bar{x}}=0\;\mathrm{at}\;\bar{x}=0\;\mathrm{and}\;{\bar{w}}_{,\bar{x}}={\bar{w}}_{,\bar{x}\bar{x}\bar{x}}=0\;\mathrm{at}\;\bar{x}=1,\;$$

(22) $$\mathrm{Case}\;H-G:\;\bar{w}={\bar{w}}_{,\bar{x}\bar{x}}=0\;\mathrm{at}\;\bar{x}=0\;\mathrm{and}\;{\bar{w}}_{,\bar{x}}={\bar{w}}_{,\bar{x}\bar{x}\bar{x}}=0\;\mathrm{at}\;\bar{x}=1.$$

From Equations (4) and (13)–(15), the light-induced expansion can be rewritten as follows, for Case I,

(23) $${\bar{\varepsilon }}_{L}\left(\bar{t}\right)=\frac{{\bar{C}}_{0}{\bar{I}}_{0}}{{\bar{I}}_{0}+1}\left\{1-\exp\left[-\left({\bar{I}}_{0}+1\right){\bar{t}}_{1}\right]\right\},\;$$

for Case II,

(24) $${\bar{\varepsilon }}_{L}\left(\bar{t}\right)=\frac{{\bar{C}}_{0}{\bar{I}}_{0}}{{\bar{I}}_{0}+1}+\left({\bar{\varepsilon }}_{\mathrm{dark}}-\frac{{\bar{C}}_{0}{\bar{I}}_{0}}{{\bar{I}}_{0}+1}\right)\exp\left[-({\bar{I}}_{0}+1){\bar{t}}_{2}\right],\;$$

and for Case III,

(25) $${\bar{\varepsilon }}_{L}\left(\bar{t}\right)={\bar{\varepsilon }}_{\mathrm{illum}}\exp\left(-{\bar{t}}_{3}\right),$$

where ${\bar{\varepsilon }}_{\mathrm{dark}}$ and ${\bar{\varepsilon }}_{\mathrm{illum}}$ are the light-induced expansions at the moment of switching from dark zone into illumination zone and from illumination zone into dark zone, respectively. Since ${\bar{t}}_{1}$, ${\bar{t}}_{2}$, and ${\bar{t}}_{3}$ are the durations of current process, light-induced expansion ${\bar{\varepsilon }}_{L}$ is process-related and time-dependent.

The well-known Euler’s buckling theory indicates that there exists a critical light-induced expansion ${\bar{\varepsilon }}_{\mathrm{cr}}$ for triggering the buckling, which is

(26) $${\bar{\varepsilon }}_{\mathrm{cr}}={\left(\pi /\mu \right)}^{2},$$

where μ is the length factor, which depends on the constraint of LCE Euler rod.

### 2.4. Solution to the Dynamic Euler Buckling of the LCE Rods

Equations (16)–(25) govern the dynamic Euler buckling of the LCE rods under steady illumination. It is usually difficult to obtain the exact solutions for the structural configuration of the rods, and the suitable approximate admissible displacement functions for the field variables can be introduced [65]. The suitable approximate admissible trigonometric functions are assumed for lateral displacement variation $\bar{w}$ of the rods in Figure 2, which satisfies the essential boundary conditions $\bar{w}=0$ and/or ${\bar{w}}_{,\bar{x}}=0$ at the ends of rods in Equations (18)–(22). Then, integrating Equation (16) with respect to $\bar{x}$, we obtain

(27) $$\bar{u}=\int [\int \left(-{\bar{w}}_{,\bar{x}}{\bar{w}}_{,\bar{x}\bar{x}}\right)+C_{1}]d\bar{x}+C_{2},$$

where $C_{1}$ and $C_{2}$ are constants of integration. As mentioned above, the rods with axially immovable ends are considered. Once the expression for $\bar{w}$ is substituted, $C_{1}$ and $C_{2}$ can be obtained by using the boundary conditions $\bar{u}=0$ at two ends. To focus on the self-sustained Euler buckling of the LCE rod under steady illumination, we only consider the first-order buckling mode in the axial direction. The admissible displacement functions [61] for the lateral displacement $\bar{w}\left(\bar{x},\bar{t}\right)$ and the axial displacement $\bar{u}\left(\bar{x},\bar{t}\right)$ satisfying the geometric boundary conditions are listed in Appendix A. $W\left(\bar{t}\right)$ in Equations (A1)–(A5) represents the dimensionless lateral displacement at the midpoint of LCE rod as shown in Figure 2, i.e., $W\left(\bar{t}\right)=w\left(0.5L,t\right)/h$. 

Inserting $\bar{w}\left(\bar{x},\bar{t}\right)$ and $\bar{u}\left(\bar{x},\bar{t}\right)$ in Equations (A1)–(A5) into Equation (17) and integrating Equation (17) over $\bar{x}$ lead to

(28) $$\bar{w}=\eta_{1}\left(\bar{x}\right)W^{3}+\eta_{2}\left(\bar{x}\right)W+\bar{\beta }\eta_{3}\left(\bar{x}\right)W_{,\bar{t}}+\eta_{3}\left(\bar{x}\right)W_{,\bar{t}\bar{t}}+C_{3}{\bar{x}}^{3}+C_{4}{\bar{x}}^{2}+C_{5}\bar{x}+C_{6},$$

where the functions $\eta_{1}\left(\bar{x}\right)$~$\eta_{3}\left(\bar{x}\right)$ are given as Equations (A6)–(A10) in Appendix A, and $W_{,\bar{t}}$ and $W_{,\bar{t}\bar{t}}$ are the first-order and second-order differentiations with respect to $\bar{t}$ respectively. The unknown integral constants $C_{3}$–$C_{6}$ are determined by the boundary conditions in Equations (18)–(22), which are presented as Equations (A11)–(A15) in Appendix A.

Substituting $\eta_{1}\left(\bar{x}\right)$~$\eta_{3}\left(\bar{x}\right)$, $C_{3}$–$C_{6}$, and $\bar{x}=0.5$ into Equation (28) yields

(29) $$W_{,\bar{t}\bar{t}}\left(\bar{t}\right)+\bar{\beta }W_{,\bar{t}}\left(\bar{t}\right)+f_{1}W\left(\bar{t}\right)+f_{2}W{\left(\bar{t}\right)}^{3}=f_{3}{\bar{\varepsilon }}_{L}\left(\bar{t}\right)W\left(\bar{t}\right),$$

The coefficients $f_{1}$, $f_{2}$, and $f_{3}$ of the LCE rod can derived from different boundary conditions and their detailed forms are listed in Appendix A.

The corresponding initial conditions of Equation (29) can be expressed as

(30) $$W=W_{0}\;\mathrm{and}\;W_{,\bar{t}}=W_{,\bar{t}0}\;\mathrm{at}\;\bar{t}=0.\;$$

It is worth noting that Equation (29) governs the dynamic Euler bucking of the LCE rod under steady illumination. Similar to Duffing oscillator [66], for $f_{1}>0$ in governing Equation (29), the LCE rod may be interpreted as a self-regulating oscillator with a spring whose restoring force is written as $F_{k}=-f_{1}W\left(\bar{t}\right)-f_{2}W{\left(\bar{t}\right)}^{3}$. ${\bar{\varepsilon }}_{L}$ is determined by the light-induced expansion at the moment of transforming between illumination and dark zone, and the duration (${\bar{t}}_{1}$, ${\bar{t}}_{2}$ or ${\bar{t}}_{3}$) of current process. Therefore, the term $f_{3}{\bar{\varepsilon }}_{L}(\bar{t})W(\bar{t})$ in Equation (29) is process-related and can be regarded as equivalent excitation force, defined as

(31) $$F_{\mathrm{in}}=f_{3}{\bar{\varepsilon }}_{L}\left(\bar{t}\right)W\left(\bar{t}\right).$$

Similarly, the equivalent damping force for the LCE rod can be defined as

(32) $$F_{\mathrm{out}}=-\bar{\beta }W_{,\bar{t}}\left(\bar{t}\right).\;$$

Self-sustained Euler buckling may arise from the competition between equivalent excitation force and equivalent damping force. 

For the differential Equation (29) with variable coefficients ${\bar{\varepsilon }}_{L}\left(\bar{t}\right)$, it can be numerically solved by combing Equations (13)–(15) and (29) based on the Runge–Kutta method. In the calculation, for the previous lateral displacement $W_{i-1}$, we can calculate the current light-induced expansion ${\bar{\varepsilon }}_{Li}$ from Equations (23)–(25). Note that the LCE rod is in the illumination zone while $\left|W_{i-1}\right|<\Delta$, and in the dark zone while $\left|W_{i-1}\right|>\Delta$. Next, based on this light-induced expansion ${\bar{\varepsilon }}_{\mathrm{Li}}$, we can further calculate the current lateral displacement $W_{i}$ from Equation (29). Then, the current ${\bar{\varepsilon }}_{L\left(i+1\right)}$ can be calculated from Equations (23)–(25) depending on the lateral displacement $W_{i}$. By iteration calculation, we can obtain the time histories of light-induced expansion and lateral displacement. 

Considering that for large ${\bar{I}}_{0}$, the light-induced expansion ${\bar{\varepsilon }}_{L}$ in Equations (23) and (24) quickly approaches to the maximum $\varepsilon_{0}$, we assume that the light-induced expansion in the narrow illumination zone Δ is constant and time-independent by treating ${\bar{\varepsilon }}_{L}\approx {\bar{\varepsilon }}_{L}\left(0\right)=\varepsilon_{0}$, where Δ is fixed to be 0.01 in the computation. 

## 3. Two Motion Modes and Their Mechanisms

Based on the governing Equations (23)–(25), (29) and (30), the dynamic Euler buckling of the LCE rod can be numerically investigated. To focus on the motion mode of system and its mechanism, we take the case H-H of five typical Euler rod as an example in this section. We first present two typical motion modes of the Euler rod: the static mode and self-buckling mode. Next, the key physical quantities in the self-sustained Euler buckling are studied in detail. Then, the corresponding mechanism of self-sustained Euler buckling is elucidated.

### 3.1. Two Motion Modes

To investigate the self-sustained Euler buckling LCE rod under steady illumination, we should determine the typical values of dimensionless parameters in the model. From the existing experiments [60,61,62], the typical material properties and geometric parameters are listed in Table 1. The corresponding dimensionless parameters are also listed in Table 2. In the following, these values of parameters are used to study the self-sustained Euler buckling LCE rod under steady illumination. 

**Figure 3:**
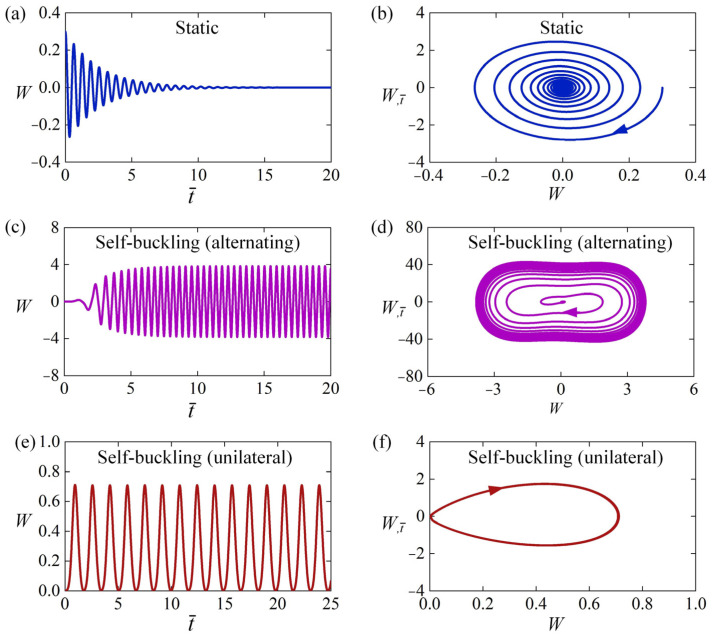
Time histories and phase trajectories of the two motion modes of the LCE rod: (**a**,**b**) The static mode for $\varepsilon_{0}=0$, $\bar{\beta }=0.8$, ${\bar{T}}_{0}=0.5$, $W_{0}=0.3$ and $W_{,\bar{t}0}=0$; (**c**,**d**) The alternating self-sustained Euler buckling mode for $\varepsilon_{0}=20$, $\bar{\beta }=0.5$, ${\bar{T}}_{0}=0.5$, $W_{0}=0$ and $W_{,\bar{t}0}=0$. (**e**,**f**) The unilateral self-sustained Euler buckling mode for $\varepsilon_{0}=40$, $\bar{\beta }=13$, ${\bar{T}}_{0}=0.5$, $W_{0}=0$ and $W_{,\bar{t}0}=0$.

Figure 3 show two typical motion modes: static mode and self-sustained Euler buckling mode. For $\varepsilon_{0}=0$, $\bar{\beta }=0.8$, ${\bar{T}}_{0}=0.5$, $W_{0}=0.3$, and $W_{,\bar{t}0}=0$, the time history curve and phase trajectory curve of the LCE rod are plotted in Figure 3a,b, respectively. The numerical results show that the amplitude of lateral displacement W gradually decreases with time from the initial position $W_{0}=0.3$ due to the damping dissipation, and the LCE rod eventually stays static, which is named as the static mode. For $\varepsilon_{0}=20$, $\bar{\beta }=0.8$, ${\bar{T}}_{0}=0.5$, $W_{0}=0$, and $W_{,\bar{t}0}=0$, the time history curve and phase trajectory curve of the vibration are plotted in Figure 3c,d, respectively. It is worth noting that the light-induced expansion loading $\varepsilon_{0}$ is larger than the critical strain ${\bar{\varepsilon }}_{\mathrm{cr}}$, which is calculated to be 9.87 from Equation (26) due to μ=1 for case H-H. The LCE rod initially buckles from the static equilibrium position and moves into the non-illuminated zone. Due to the decrease of light-induced expansion in the non-illuminated zone with time, the rod tends to move back to the illumination zone again. Then, it may buckle to another side of the static equilibrium position due to its own inertia. Eventually, the LCE rod under steady illumination may evolve into a continuous alternating buckling in both sides of the static equilibrium position, which is named as self-buckling mode. It is worth mentioning that similar experiment works about light-driven buckling of LCE have been reported in Refs. [31,32,33]. 

It should be noted that the LCE rod under steady illumination may unilaterally self-buckle on one side of the static equilibrium position, as shown in Figure 3e,f. For $\varepsilon_{0}=40$, $\bar{\beta }=13$, ${\bar{T}}_{0}=0.5$, $W_{0}=0$, and $W_{,\bar{t}0}=0$, the time history curve and phase trajectory curve of the vibration are plotted in Figure 3e,f, respectively. It can be clearly seen that the rod self-buckles continuously and unilaterally from the initial static state. This is because that the velocity of the rod drops to zero before it moves back to static equilibrium position, and when it is illuminated in the narrow illumination zone again, it buckles in the same side as the previous buckling. Considering that the mechanisms of alternating self-sustained Euler buckling and unilateral self-sustained Euler buckling are similar, we mainly focus on the alternating self-sustained Euler buckling in the following discussion.

### 3.2. Evolution of Key Physical Quantities

**Figure 4:**
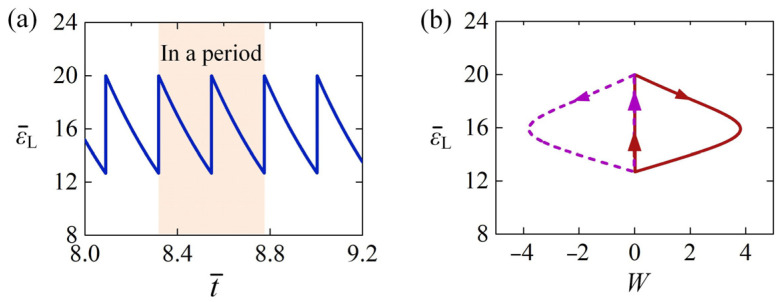
(**a**) The time history of light-induced expansion of the rod shown in Figure 3c,d. (**b**) The dependence of light-induced expansion on lateral displacement in one cycle of self-sustained Euler buckling. The light-induced expansion of the rod changes periodically with time during the dynamic Euler buckling.

Several key physical quantities in the LCE rod under steady illumination vary with time during the dynamic buckling, and they are calculated in the following part for the typical case in Figure 3c,d. Figure 4a shows the time history of the light-induced expansion ${\bar{\varepsilon }}_{L}$, presenting the characteristics of periodic changes over time. Figure 4b plots its phase trajectory between the light-induced expansion ${\bar{\varepsilon }}_{L}$ and lateral displacement W of the LCE rod in one cycle of the self-sustained Euler buckling. The red solid and purple dotted curves in Figure 4b correspond to the vibration process of the rod buckling along the positive and negative directions of *z*-axis respectively. Initially, the LCE rod is in the illumination zone, and the light-induced expansion ${\bar{\varepsilon }}_{L}$ greatly increases with time. Then, the LCE rod moves away from the static equilibrium position to the non-illumination zone, and the light-induced expansion ${\bar{\varepsilon }}_{L}$ undergoes a gradual decrease with time in the dark. When the LCE rod moves back and passes through the static equilibrium position being irradiated again, the light-induced expansion ${\bar{\varepsilon }}_{L}$ greatly increases. Subsequently, the rod buckles to another side of the static equilibrium position. Eventually, the LCE rod vibrates periodically and alternately in both sides of the static equilibrium position. In one vibration cycle, the light-induced expansion ${\bar{\varepsilon }}_{L}$ forms a symmetrical closed loop in Figure 4b. 

**Figure 5:**
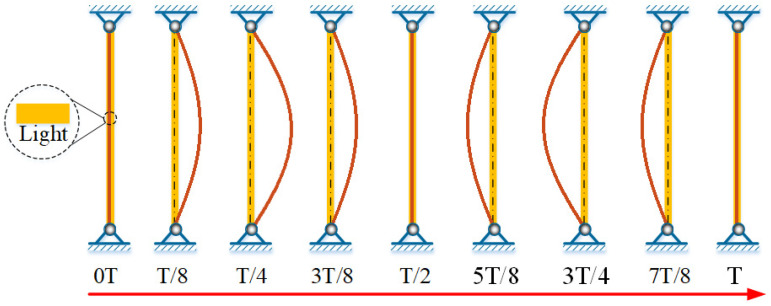
The snapshots in one cycle of self-sustained Euler buckling for the LCE rod shown in Figure 3c,d. The LCE rod under steady illumination self-sustained Euler buckles periodically and alternating due to the periodically varying light-induced expansion.

Figure 5 shows the snapshots in one cycle of self-sustained Euler buckling of the LCE rod shown in Figure 3c,d, where T is the vibration period. Clearly observing that with the increase or decrease of the lateral displacement W at the midpoint of the rod, the lateral displacement $\bar{w}\left(\bar{x}\right)$ at each position of the rod also increases or decreases gradually. This result can be easily interpreted by Equation (A1) in Appendix A that $\bar{w}\left(\bar{x}\right)$ is jointly determined by the lateral displacement W at the midpoint of the rod and the axial position $\bar{x}$. 

**Figure 6:**
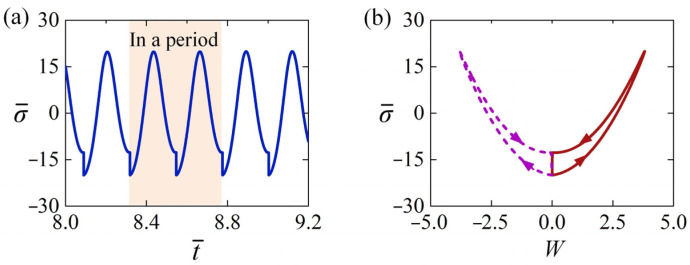
(**a**) The time history of normal stress of the rod shown in Figure 3c,d. (**b**) The dependence of normal stress on lateral displacement in a cycle of Euler buckling. The normal stress of the rod varies periodically during dynamic Euler buckling.

Figure 6a presents the time history of normal stress σ of the LCE rod shown in Figure 3c,d, where the normal stress σ is nondimensionalized as $\bar{\sigma }=\sigma AL^{2}/EJ$. The diagram reveals that the normal stress varies periodically with time. Figure 6b plots its phase trajectory between the normal stress $\bar{\sigma }$ and the lateral displacement W in one cycle of the self-sustained Euler buckling. The red curve corresponds to the dynamic buckling along the positive direction of *z*-axis. In this paper, the rod is tensile for positive normal stress $\bar{\sigma }$, while it is compressive for negative $\bar{\sigma }$. It is shown that the absolute value of negative normal stress $\bar{\sigma }$, i.e., compressive normal stress sharply increases to maximum, due to the great increase of light-induced expansion ${\bar{\varepsilon }}_{L}$ in illumination zone. This means that the compressive stress reaches its maximum at the static equilibrium position, which leads to the buckling of the rod. Next, the rod moves away from the static equilibrium position, the normal stress gradually increases and then becomes positive. The exact reason for such result is that the light-induced expansion ${\bar{\varepsilon }}_{L}$ decreases with time in the dark, and the nonlinear deformation strain ${\bar{\varepsilon }}_{x}=\varepsilon_{x}AL^{2}/J$ increases with the increased W.

Then, the LCE rod gradually moves back to the static equilibrium position, and the tension is gradually relaxed, resulting from the continual decrease of light-induced expansion ${\bar{\varepsilon }}_{L}$ and nonlinear deformation strain ${\bar{\varepsilon }}_{x}$. While the rod returns to the static equilibrium position and passes through the narrow illumination zone again, the normal stress behaves a sharp decay because of the rapidly increasing light-induced expansion in illumination. Thereafter, the rod continues to move along the negative direction of *z*-axis due to its inertia. Its normal stress also firstly increases and then decays with time in the dark. Then, the rod moves back the static equilibrium position again and finally vibrates periodically. In a vibration cycle, the normal stress $\bar{\sigma }$ of the rod also varies periodically and forms a symmetrical closed loop in Figure 6b.

### 3.3. Mechanism of the Self-Sustained Euler Buckling 

To further explore the self-sustained Euler buckling mechanism of the LCE rod, we investigate the competition between energy input and energy dissipation, which can be described by the work done by equivalent excitation force $F_{\mathrm{in}}$ in Equation (31) and equivalent damping force $F_{\mathrm{out}}$ in Equation (32), respectively. There is no analytic solution for the governing differential Equation (29) with variable coefficients ${\bar{\varepsilon }}_{L}\left(\bar{t}\right)$, so we conduct the numerical calculations to compare the energy input and energy dissipation.

**Figure 7:**
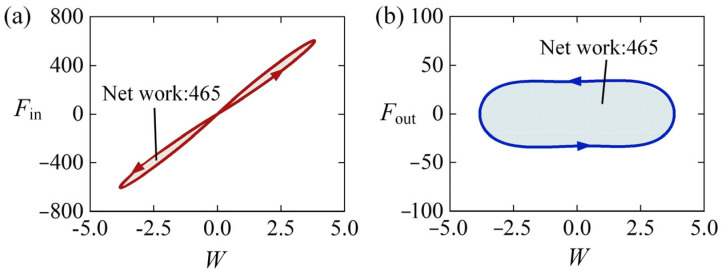
The mechanism of self-sustained Euler buckling of the LCE rod shown in Figure 3c,d. (**a**) $F_{\mathrm{in}}$ vs. W; (**b**) $F_{\mathrm{out}}$ vs. W. During the self-sustained Euler buckling of the LCE rod, the damping dissipation from the system equals to the work done by the excitation force.

Figure 7a shows the dependence of the equivalent excitation force on different lateral displacement W in one cycle of the self-sustained Euler buckling of the rod shown in Figure 3c,d. In the process of dynamic Euler buckling, we observe a gradual increase in the equivalent excitation force $F_{\mathrm{in}}$ as the LCE rod moves away from the static equilibrium position. Similarly, it decreases gradually as the rod moves back to the static equilibrium position. Therefore, the dependence between the equivalent excitation force $F_{\mathrm{in}}$ and the lateral displacement W presents a closed clockwise curve, and the red shadow area in Figure 7a implies the positive net work done by the equivalent excitation force $F_{\mathrm{in}}$. In fact, the net work originates from the elastic energy during the dynamic Euler buckling of the LCE rod. As shown in Figure 7b, the dependence between the equivalent damping force $F_{\mathrm{out}}$ and the lateral displacement W presents a closed counterclockwise curve, implying that $F_{\mathrm{out}}$ does negative net work which is exactly equal to the net work done by the excitation force $F_{\mathrm{in}}$. Hence, during the self-sustained Euler buckling of the LCE rod, the damping dissipation from the system is compensated by the net work done by the equivalent excitation force, and thus the LCE rod under steady illumination can self-buckle continuously and alternately in both sides of the static equilibrium position. 

## 4. Influence of System Parameters

In this section, we explore the effects of physical quantities on the triggering conditions, the frequency and amplitude of the self-sustained Euler buckling of the rod. In this work, we take case H-H as an example and focus on the influence of two typical physical parameters of the system: light-induced expansion loading $\varepsilon_{0}$ and initial condition $W_{0}$. In the following discussion, f denotes the dimensionless frequency, and A denotes the dimensionless amplitude which is the maximum value of W at midpoint of rod.

### 4.1. Effect of the Initial Condition

**Figure 8:**
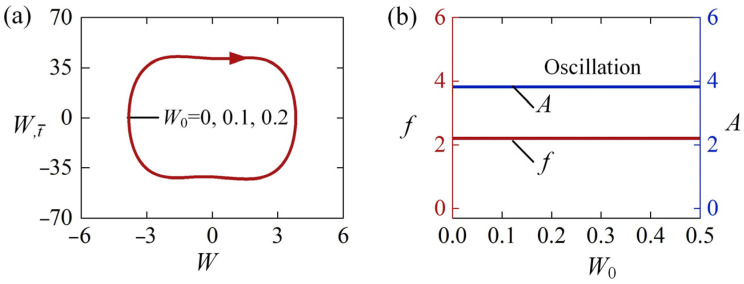
The effect of initial condition $W_{0}$ on the self-sustained Euler buckling of the LCE rod, for $\varepsilon_{0}=20$, $\bar{\beta }=0.8$, ${\bar{T}}_{0}=0.5$, and $W_{,\bar{t}0}=0$. (**a**) Limit cycles. (**b**) Frequency and amplitude. The initial condition has no effect on the self-sustained Euler buckling of the LCE rod.

Figure 8 shows the effect of $W_{0}$ on the self-sustained Euler buckling of the LCE rod for the case of H-H. In the calculation, we set $\varepsilon_{0}=20$, $\bar{c}=0.8$, ${\bar{T}}_{0}=0.5$, and $W_{,\bar{t}0}=0$. Figure 8a plots the limit circles of the self-sustained Euler buckling rod for three different initial positions $W_{0}=0$, $W_{0}=0.1$, and $W_{0}=0.2$. The detailed numerical results demonstrate that the limit cycles of the self-sustained Euler buckling are identical for different $W_{0}$. Figure 8b plots the frequency and amplitude of the self-sustained Euler buckling as a function of the initial position $W_{0}$. It can be easily observed that the frequency and amplitude of the self-sustained Euler buckling do not vary with the increase of $W_{0}$. Considering that the initial velocity $W_{,\bar{t}0}=0$ is related to the initial position $W_{0}$ by energy conversion, it can be concluded that the initial condition does not affect the self-sustained Euler buckling of the LCE rod. This is because the self-sustained Euler buckling of the rod results from the energy competition between the damping dissipation and net work done by the excitation force, and the frequency and amplitude are determined by the intrinsic characteristics of the system, which is consistent with the general characteristics of other self-oscillating systems [1]. 

### 4.2. Effect of the Light-Induced Expansion Loading

**Figure 9:**
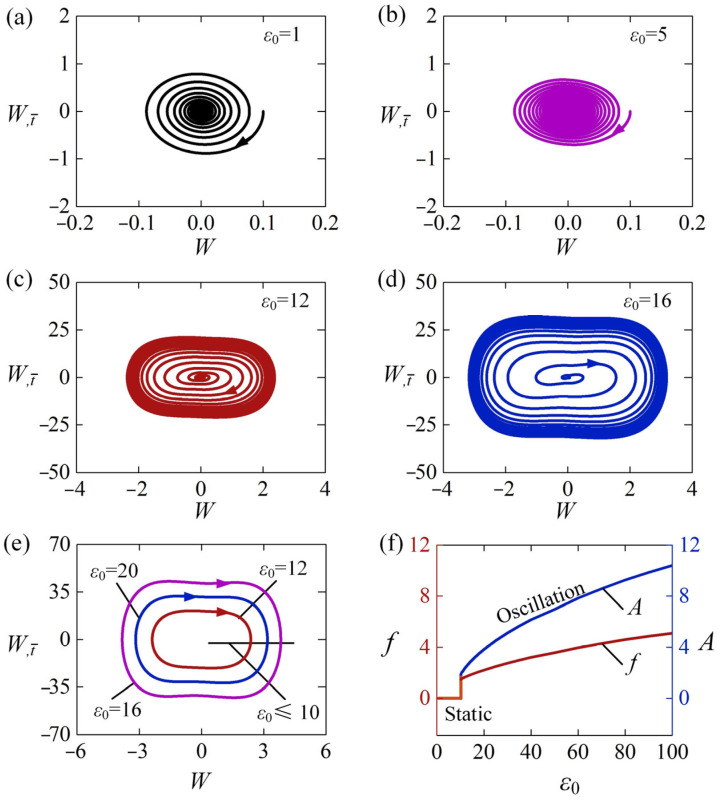
The effect of light-induced expansion loading $\varepsilon_{0}$ on the self-sustained Euler buckling of the LCE rod, for $\bar{\beta }=0.8$, ${\bar{T}}_{0}=0.5$, $W_{0}=0.1$, and $W_{,\bar{t}0}=0$. Phase trajectories of the self-sustained Euler buckling mode for (**a**) $\varepsilon_{0}=1$, (**b**) $\varepsilon_{0}=5$, (**c**) $\varepsilon_{0}=12$, and (**d**) $\varepsilon_{0}=16$, (**e**) Limit cycles. (**f**) Frequency and amplitude. A critical $\varepsilon_{0}\approx 10$ for phase transition between static mode and self-sustained Euler buckling mode is found.

Figure 9 presents the effect of light-induced expansion loading on the self-sustained Euler buckling of the rod with the boundary configuration H-H, for $\bar{\beta }=0.8$, ${\bar{T}}_{0}=0.5$, $W_{0}=0.1$, and $W_{,\bar{t}0}=0$. Figure 9a,b plot the phase trajectories of static mode for $\varepsilon_{0}=1$ and $\varepsilon_{0}=5$, respectively. For the vibration velocity at the midpoint of the rod, we witness an initial increase, and then a gradual decrease until the vibration stops. This is because for small $\varepsilon_{0}$, the net work done by the equivalent excitation force transformed from the constant illumination is small, and not enough to compensate for the damping dissipation. Figure 9c,d plot the phase trajectories of the self-sustained Euler buckling for $\varepsilon_{0}=12$ and $\varepsilon_{0}=16$, respectively. It can be easily observed that the self-sustained Euler buckling can be triggered for the two light-induced expansion loadings. From the limit cycles plotted in Figure 9e, there is a critical $\varepsilon_{0}$ for the phase transition between the static mode and the self-sustained Euler buckling mode, which is numerically calculated to be about 10. For $\varepsilon_{0}\leq 10$, the LCE rod keeps in the static mode and is unable to self-buckle. Figure 9f describes the dependences of frequency and amplitude on $\varepsilon_{0}$ for the self-sustained Euler buckling. It is clearly seen that both frequency and amplitude of the self-sustained Euler buckling present upward trends with the increasing $\varepsilon_{0}$. This is due to the mechanical energy converted from the light energy increasing along with the increase of $\varepsilon_{0}$. With consideration of $\varepsilon_{0}=C_{0}{\bar{I}}_{0}L^{2}A/\left[\left({\bar{I}}_{0}+1\right)J\right]$, the result means that the increase of slenderness ratio or light-induced expansion coefficient is capable of promoting the self-sustained Euler buckling of the LCE rod. 

## 5. Self-Buckling Mode: Different Boundary Conditions

Different from the general self-oscillating systems, the boundary configuration of the Euler rod is easily modulated to satisfy the requirements in various fields of applications. Therefore, it is of great significance to study the influence of boundary configuration on the self-sustained Euler buckling of the LCE rod. In this section, the effects of boundary configuration including H-H, C-C, C-H, C-G, and H-G on several important physical quantities are extensively investigated.

### 5.1. Normal Stress for Different Constraint Configurations

**Figure 10:**
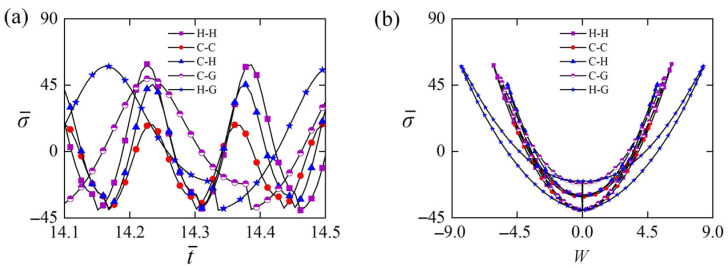
The normal stress of the LCE rod for different constraint configurations. In the computation, $\varepsilon_{0}=40$, $\bar{\beta }=0.8$, ${\bar{T}}_{0}=0.5$, $W_{0}=0.1$, and $W_{,\bar{t}0}=0$ are set. (**a**) The time histories of the normal stress. (**b**) Dependence of the normal stress $\bar{\sigma }$ on the lateral displacement W in one cycle.

Figure 10 shows the normal stress of the LCE rod for different constraint configurations. In the computation, $\varepsilon_{0}=40$, $\bar{\beta }=0.8$, ${\bar{T}}_{0}=0.5$, $W_{0}=0.1$, and $W_{,\bar{t}0}=0$ are set. The time histories of the normal stress in one cycle of self-buckling for five typical Euler rods are given in Figure 10a. Results indicate that their normal stresses $\bar{\sigma }$ vary periodically with time. Their maximum normal stresses $\bar{\sigma }$ are listed in Table 1, and can be arranged from small to large as follows: case C-C, case C-H, case C-G, case H-G, case H-H. This result demonstrates that the maximum normal stress of LCE rod generally increases with the relaxation of constraint. Figure 10b plots their dependences of the normal stress $\bar{\sigma }$ on the lateral displacement W in one cycle of the self-buckling. By comparing the three cases H-H, C-H, and C-C, it is found that their maximum normal stresses $\bar{\sigma }$ and lateral displacements W both increase with the released rotation constraint at one rod end, which can also be revealed from the comparison between case C-G and case H-G. The increase of the maximum normal stress and lateral displacement can also be achieved by releasing the movement constraint. Comparing case C-H and case C-G with C-C respectively, the maximum normal stress of loosening the movement constraint is found to be slightly larger than that of loosening the rotation constraint.

### 5.2. Equivalent Force for Different Constraint Configurations

**Figure 11:**
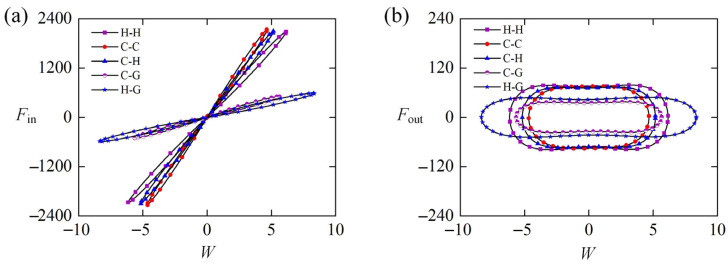
The equivalent force of the LCE rod for different constraint configurations. In the computation, $\varepsilon_{0}=40$, $\bar{\beta }=0.8$, ${\bar{T}}_{0}=0.5$, $W_{0}=0.1$, and $W_{,\bar{t}0}=0$ are set. (**a**) Dependence of the equivalent excitation force $F_{\mathrm{in}}$ on the lateral displacement W in one cycle. (**b**) Dependence of the equivalent damping force $F_{\mathrm{out}}$ on the lateral displacement W in one cycle.

Figure 11 gives the equivalent force of the LCE rod in Equations (31) and (32) for different constraint configurations. In the computation, $\varepsilon_{0}=40$, $\bar{\beta }=0.8$, ${\bar{T}}_{0}=0.5$, $W_{0}=0.1$, and $W_{,\bar{t}0}=0$ are set. Figure 11a diagrams the dependence of the equivalent excitation force $F_{\mathrm{in}}$ on the lateral displacement W in one cycle of the self-buckling, which is affected by constraint configurations. For the five typical Euler rods shown in Figure 2, their positive net work done by the equivalent excitation force can be presented by the areas enclosed by the five closed curves as shown in Figure 11a, respectively. By careful calculation, their maximum equivalent excitation forces $F_{\mathrm{in}}$, equivalent damping forces $F_{\mathrm{out}}$, and net works done by $F_{\mathrm{in}}$ ($F_{\mathrm{out}}$) are listed in Table 3. For the three cases of H-H, C-H, and C-C, the net work done by equivalent excitation force of the case H-H is maximum. It can be understood that the net work done by excitation force can be increased by releasing the constraint at the rod end. Comparing with case C-C, the movement constraint of case C-G and the rotation constraint of case C-H are released, respectively. Meanwhile, the net work of case C-G done by equivalent excitation force is larger than that of case C-H as shown in Table 3, for relaxing the movement constraint can increase much more net work done by the equivalent excitation force than relaxing the rotation constraint. 

Figure 11b shows the dependence of the equivalent damping force $F_{\mathrm{out}}$ on the lateral displacement W in one cycle for five typical Euler rods shown in Figure 2. In each case, the net work done by the equivalent damping force, denoted by the area surrounded by the equivalent damping force and lateral displacement curve, is equal to the net work done by the corresponding excitation force through careful calculation as shown in Table 1. For the three cases of H-H, C-H, and C-C, the net work done by the equivalent damping force of case H-H is the maximum one, as shown in Figure 11b. The exact reason for such result is that the maximums of its equivalent damping force and lateral displacement W are always the largest. Comparing case C-H with case C-G, the net work done by the equivalent damping force of case C-G is more than that of case C-H, resulting from the larger maximum of its equivalent damping force. Referring to case C-C, it is also found that compared to relaxing the rotation constraint, relaxing the movement constraint can increase the net work done by the equivalent damping force more. 

### 5.3. Frequency and Amplitude for Different Constraint Configurations 

**Figure 12:**
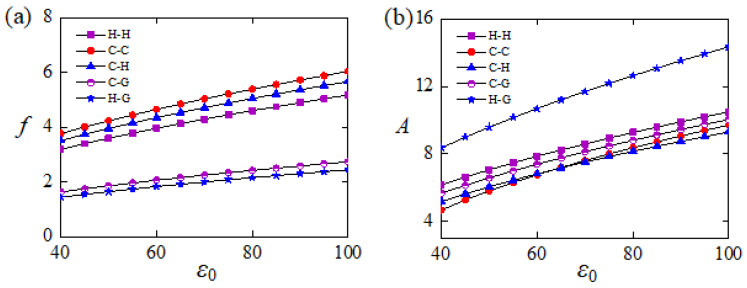
(**a**) Frequency and (**b**) amplitude of the self-buckling for different constraint configuration and light-induced expansion loading. In the computation, $\bar{\beta }=0.8$, ${\bar{T}}_{0}=0.5$, $W_{0}=0.1$, and $W_{,\bar{t}0}=0$ are set. Generally, strengthening the boundary constraint can increase the frequency and reduce the amplitude.

Figure 11 plots the frequency and amplitude of the self-buckling for different constraint configuration and light-induced expansion loading. In the computation, $\bar{\beta }=0.8$, ${\bar{T}}_{0}=0.5$, $W_{0}=0.1$, and $W_{,\bar{t}0}=0$ are set. For all the five typical constraint configurations of the Euler rods shown in Figure 2, both their frequencies and amplitudes of the self-sustained Euler buckling increase along with the increase of $\varepsilon_{0}$. As discussed in detail above, this is because increasing $\varepsilon_{0}$ can always increase the mechanical energy converted from light energy. For the given $\varepsilon_{0}$, the frequency and amplitude of LCE rods with different constraint configurations are different as shown in Figure 12. Comparing case H-H, case C-H, and case C-C in Figure 12a, it is easy to find that the frequency of case C-C is the largest one and that of case H-H is the smallest one. This implies that strengthening the constraint of LCE rod can increase the frequency of self-sustained Euler buckling. In addition, comparing case C-H and case C-G with case C-C, the frequency of case C-H is higher than that of case C-G as shown in Figure 12a. Results demonstrate that relaxing the rotation constraint can increase the frequency of self-sustained Euler buckling more, compared to relaxing the movement constraint more. 

For a given $\varepsilon_{0}$, Figure 12b provides the amplitude of five typical Euler rods with different constraint configurations shown in Figure 2. Among the case H-H, case C-H, and case C-C, the amplitude of case H-H is the highest as presented in Figure 12b, which implies that the amplitude of self-sustained Euler buckling can be increased by relaxing the constraint of LCE rod. Contrasting case C-H and case C-G with case C-C in Figure 12b, the amplitude of case C-G is greater than that of case C-H, which means that relaxing the movement constraint can increase the amplitude of self-sustained Euler buckling more dramatically than relaxing the rotation constraint. Therefore, both frequency and amplitude of the self-buckling of LCE rods can be influenced by their constraint configurations, and a variety of applications can be satisfied by adjusting the boundary configuration of the Euler rod. 

## 6. Conclusions

Euler buckling of a rod can rapidly release elastic strain energy during buckling and has potential applications for high energy release rate and remote mechanical control. Especially, the boundary configuration of Euler rod is usually easy to be modulated. In this study, we develop a self-sustained Euler buckling system based on an optically responsive LCE rod with different boundary constraints. The buckling of LCE rod results from the light-induced expansion and compressive force, and the self-buckling is maintained by the energy competition between the damping dissipation and the net work done by the effective elastic force. Based on dynamic LCE model, the governing equations for dynamic Euler buckling of the LCE rod is formulated, and approximate admissible trigonometric functions and Runge–Kutta method are used to numerically solve the dynamic Euler buckling. The analysis of results shows that there are two types of motion modes of the Euler rod: the static mode and the self-buckling mode, including alternating and unilateral self-buckling modes. The triggering conditions, frequency, and amplitude of the self-sustained Euler buckling can be modulated by several system parameters and boundary constraints. In general, strengthening the boundary constraint can increase the frequency and reduce the amplitude. It is noted that fatigue of continuous oscillating LCEs is relevant for actuators and deserves further exploration in the future. It is also worthwhile further illustrating the self-buckling rod in practice and exploring its application, and we hope that the current work may open new avenues for energy harvesters, signal sensors, mechano-logistic devices, and autonomous robots.

## Figures and Tables

**Table 1:** Material properties and geometric parameters.

Parameter	Definition	Value	Units
$C_{0}$	Contraction coefficient	0–0.5	/
$T_{0}$	*trans*-to-*cis* thermal relaxation time	0.1	s
$I_{0}$	Light intensity	0–1	kW/m^2^
$\eta_{0}$	Light-absorption constant	0.0003	m^2^/(s∙W)
E	Elastic modulus of LCE balloon	1–10	MPa
ρ	Mass density	10^3^	kg/m^3^
L	Length of LCE rod	10–50	mm
A	Cross-sectional area of LCE rod	0–0.1	mm^2^
J	Area moment of inertia of LCE rod	(0–1) × 10^−6^	mm^4^
β	Damping coefficient	0–0.001	mg∙mm^2^/s

**Table 2:** Dimensionless parameters.

Parameter	$W_{0}$	$W_{,\bar{t}0}$	$\varepsilon_{0}$	$\bar{\beta }$	${\bar{T}}_{0}$
Value	0–0.5	0–1	0–100	0–0.1	0–2

**Table 3:** Several key physical quantities for different boundary conditions.

	Maximum W(w(0.5L,t)/h)	$\mathrm{Maximum}\;\bar{\sigma }$ $\left(\sigma AL^{2}/\mathrm{EJ}\right)$	$\mathrm{Maximum}\;F_{\mathrm{in}}\;(\mathrm{Equation}\;(31))$	$\mathrm{Maximum}\;F_{\mathrm{out}}\;(\mathrm{Equation}\;(32))$	$\mathrm{Work}\;\mathrm{Done}\;\mathrm{by}\;F_{\mathrm{in}}$ $\;(F_{\mathrm{out}})$
Case H-H	6.16	59.41	2084.97	78.72	1761
Case C-C	4.67	18.91	2139.81	75.72	1254
Case C-H	5.18	45.59	2099.59	73.75	1393
Case C-G	5.66	49.64	506.85	37.40	754
Case H-G	8.36	57.98	590.86	48.60	1435

## Data Availability

The data that support the findings of this study are available upon reasonable request from the authors.

## Appendix A

The admissible displacement functions for the lateral displacement $\bar{w}\left(\bar{x},\bar{t}\right)$ and the axial displacement $\bar{u}\left(\bar{x},\bar{t}\right)$ are

(A1) $$\mathrm{Case}\;H-H:\;\bar{w}=W\sin\left(\pi \bar{x}\right)\;\mathrm{and}\;\bar{u}=-\frac{W^{2}\pi }{8}\sin\left(2\pi \bar{x}\right),\;$$

(A2) $$\mathrm{Case}\;C-C:\;\bar{w}=\frac{W}{2}\left[1-\cos\left(2\pi \bar{x}\right)\right]\;\mathrm{and}\;\bar{u}=\frac{W^{2}}{16}\pi \sin\left(4\pi \bar{x}\right),\;$$

(A3) $$\mathrm{Case}\;C-H:\;\bar{w}=0.1709382933\;W\left[\sin\left(k\bar{x}\right)-k\cos\left(k\bar{x}\right)+k\left(1-\bar{x}\right)\right]\;\mathrm{and}\;\bar{u}=W^{2}\left[\begin{array}{l} 0.3159893170\sin\left(2k\bar{x}\right)+0.1478029823\cos\left(2k\bar{x}\right)+0.1314349783\sin\left(k\bar{x}\right)\;\\ -0.5912119294\cos\left(k\bar{x}\right)-0.4375437143\bar{x}+0.4434089319 \end{array}\right],$$

(A4) $$\mathrm{Case}\;C-G:\;\bar{w}=W\left[1+\cos\left(\pi \bar{x}\right)\right]\;\mathrm{and}\;\bar{u}=\frac{W^{2}}{8}\pi \sin\left(2\pi \bar{x}\right),\;$$

(A5) $$\mathrm{Case}\;H-G:\;\bar{w}=\sqrt{2}W\sin\left(\frac{\pi \bar{x}}{2}\right)\;\mathrm{and}\;\bar{u}=-\frac{W^{2}}{8}\pi \sin\left(\pi \bar{x}\right),$$

where $W\left(\bar{t}\right)$ is the dimensionless lateral displacement at the midpoint of LCE rod.

The functions $\eta_{1}\left(\bar{x}\right)$~$\eta_{3}\left(\bar{x}\right)$ are

(A6) $$\mathrm{Case}\;H-H:\;\eta_{1}=-\frac{\sin\left(\pi \bar{x}\right)}{4},\;\eta_{2}=\frac{{\bar{\varepsilon }}_{L}\sin\left(\pi \bar{x}\right)}{\pi^{2}}\;\mathrm{and}\;\eta_{3}=-\frac{\sin\left(\pi \bar{x}\right)}{\pi^{4}},\;$$

(A7) $$\mathrm{Case}\;C-C:\;\eta_{1}=\frac{\cos\left(2\pi \bar{x}\right)}{32},\;\eta_{2}=-\frac{{\bar{\varepsilon }}_{L}\cos\left(2\pi \bar{x}\right)}{8\pi^{2}}\;\mathrm{and}\;\eta_{3}=\frac{\cos\left(2\pi \bar{x}\right)}{32\pi^{4}}-\frac{{\bar{x}}^{4}}{48},$$

(A8) $$\mathrm{Case}\;C-H:\;\eta_{1}=0.116649\;\sin\left(k\bar{x}+1.78955\right),\;\eta_{2}=-0.0389298\;\sin\left(k\bar{x}+1.78955\right)\;\mathrm{and}\;\;\eta_{3}=0.0001924058\sin\left(k\bar{x}+1.78955\right)-0.032037627{\bar{x}}^{4}+0.006407525{\bar{x}}^{5},$$

(A9) $$\mathrm{Case}\;C-G:\;\eta_{1}=-\frac{\cos\left(\pi \bar{x}\right)}{4},\;\eta_{2}=\frac{{\bar{\varepsilon }}_{L}\cos\left(\pi \bar{x}\right)}{\pi^{2}}\;\mathrm{and}\;\eta_{3}=\frac{\cos\left(\pi \bar{x}\right)}{\pi^{4}}-\frac{x^{4}}{24},\;$$

(A10) $$\mathrm{Case}\;H-G:\;\eta_{1}=-\frac{\sqrt{2}\sin\left(\pi \bar{x}/2\right)}{2},\;\eta_{2}=\frac{4\sqrt{2}{\bar{\varepsilon }}_{L}\sin\left(\pi \bar{x}/2\right)}{\pi^{2}}\mathrm{and}\;\eta_{3}=-\frac{16\sqrt{2}\sin\left(\pi \bar{x}/2\right)}{\pi^{4}}.\;$$

The integral constants $C_{3}$~$C_{6}$ are

(A11) $$\mathrm{Case}\;H-H:\;C_{3}=0,\;C_{4}=0,\;C_{5}=0,\;\mathrm{and}\;C_{6}=0,$$

(A12) $$\mathrm{Case}\;C-C:\;C_{3}=\frac{\left(W_{,\bar{t}\bar{t}}+\bar{\beta }W_{,\bar{t}}\right)}{24},\;C_{4}=\frac{-C_{3}}{2},\;C_{5}=0,\;\;\;\;\;\;\;\;\;\;\;\;\;\;\;\;\;\;\;\;\;\;\;\;\;\;\;\;\mathrm{and}\;C_{6}=\frac{-\left(W_{,\bar{t}\bar{t}}+\bar{\beta }W_{,\bar{t}}+\pi^{4}W^{3}-{4\pi }^{2}W{\bar{\varepsilon }}_{L}\right)}{32\pi^{4}},$$

(A13) $$\mathrm{Case}\;C-H:\;C_{3}=0.00291777W^{3}+0.05130833W_{,\bar{t}\bar{t}}+0.05130833\bar{\beta }W_{,\bar{t}}-0.00097376W{\bar{\varepsilon }}_{L},\;C_{4}=-0.00344271W^{3}-0.02568689W_{,\bar{t}\bar{t}}-0.02568689\bar{\beta }W_{,\dot{t}}+0.00114895W{\bar{\varepsilon }}_{L},\;C_{5}=0.11386905W^{3}+0.00187820W_{,\bar{t}\bar{t}}+0.00187820\bar{\beta }W_{,\bar{t}}-0.03800206W{\bar{\varepsilon }}_{L},\;\mathrm{and}\;C_{6}=-0.11386905W^{3}-0.00187820W_{,\bar{t}\bar{t}}-0.00187820\bar{\beta }W_{,\bar{t}}+0.03800206W{\bar{\varepsilon }}_{L}.\;$$

(A14) $$\mathrm{Case}\;C-G:\;C_{3}=C_{5}=0,\;C_{4}=\frac{\left(W_{,\bar{t}\bar{t}}+\bar{\beta }W_{,\bar{t}}\right)}{12},\;\;\mathrm{and}\;C_{6}=-\frac{\left(24+\pi^{2}\right)\left(W_{,\bar{t}\bar{t}}+\bar{\beta }W_{,\bar{t}}\right)}{24\pi^{4}}-\frac{\pi^{2}W^{3}-4{\bar{\varepsilon }}_{L}W}{4\pi^{2}},$$

(A15) $$\mathrm{Case}\;H-G:\;C_{3}=0,\;C_{4}=0,\;C_{5}=0,\;\mathrm{and}\;C_{6}=0.\;$$

The coefficients $f_{1}$, $f_{2}$, and $f_{3}$ are

(A16) $$\mathrm{Case}\;H-H:\;f_{1}=\pi^{4},\;f_{2}=\frac{\pi^{4}}{4},\;\mathrm{and}\;f_{3}=\pi^{2},\;$$

(A17) $$\mathrm{Case}\;C-C:\;f_{1}=\frac{768\pi^{4}}{48+\pi^{4}},\;f_{2}=\frac{48\pi^{4}}{48+\pi^{4}},\;\mathrm{and}\;f_{3}=\frac{192\pi^{2}}{48+\pi^{4}},$$

(A18) $$\mathrm{Case}\;C-H:\;f_{1}=235.128010,\;f_{2}=34.937392,\;\mathrm{and}\;f_{3}=11.659822,$$

(A19) $$\mathrm{Case}\;C-G:\;f_{1}=\frac{128\pi^{4}}{128+3\pi^{4}},\;f_{2}=\frac{32\pi^{4}}{128+3\pi^{4}},\;\mathrm{and}\;f_{3}=\frac{128\pi^{2}}{128+3\pi^{4}},$$

(A20) $$\mathrm{Case}\;H-G:\;f_{1}=\frac{\pi^{4}}{16},\;f_{2}=\frac{\pi^{4}}{32},\;\mathrm{and}\;f_{3}=\frac{\pi^{2}}{4}.$$
